# Harsh discipline relates to internalizing problems and cognitive functioning: findings from a cross-sectional study with school children in Tanzania

**DOI:** 10.1186/s12888-016-0828-3

**Published:** 2016-04-29

**Authors:** Tobias Hecker, Katharin Hermenau, Charlotte Salmen, Martin Teicher, Thomas Elbert

**Affiliations:** Department of Psychology, University of Zurich, Binzmuehlestr. 14/17, 8050 Zurich, Switzerland; Department of Psychology, University of Konstanz, Box 905, 78457 Konstanz, Germany; vivo international, Box 5108, 78430 Konstanz, Germany; Department of Psychiatry, Harvard Medical School, 401 Park Drive, 02215 Boston, MA USA; Developmental Biopsychiatry Research Program, McLean Hospital, 115 Mill Street, 02478 Belmont, MA USA

**Keywords:** Harsh discipline, Mental health, Internalizing problems, Working memory, School performance

## Abstract

**Background:**

Child maltreatment poses a risk to children and adolescents’ mental health and may also affect cognitive functioning. Also harsh discipline has been frequently associated with mental health problems. However, within societies in which harsh disciplinary methods are culturally normed and highly prevalent less is known about the association between harsh punishment, mental health problems, and cognitive functioning.

**Methods:**

In a cross-sectional study, we conducted structured clinical interviews with a sample of Tanzanian primary school students assessing exposure to harsh discipline (Maltreatment and Abuse Chronology of Exposure), internalizing problems (Strength and Difficulties Questionnaire, Children’s Depression Inventory), and working memory (Corsi Blocktapping Task). School performance was measured by using the exam grades in 4 core subjects. The 409 children (52 % boys) had a mean age of 10.5 years (range: 6 – 15).

**Results:**

Using structural equation modeling, a strong relationship was found between harsh discipline and internalizing problems (β = .47), which were related to lower working memory capacity (β = −.17) and school performance (β = −.17).

**Conclusions:**

The present study suggests that harsh discipline is closely linked to children’s internalizing mental health problems, which are in turn associated with lower cognitive functioning and school performance. Given the high rates of harsh discipline experienced by children in East African homes and elsewhere, the findings of the present study emphasize the need to inform the population at large about the potentially adverse consequences associated with harsh discipline.

**Electronic supplementary material:**

The online version of this article (doi:10.1186/s12888-016-0828-3) contains supplementary material, which is available to authorized users.

## Background

In many countries worldwide, children are frequently exposed to harsh discipline such as spanking or being beaten with objects like sticks or belts [1, 2]. We define harsh discipline as the use of any physical or psychological force with the intention of causing physical or emotional pain for the purpose of correction or control of the child’s behavior. As physical and psychological discipline occasionally harms the child but poses at least a continuous threat or stressor to the child, harsh discipline is commonly defined as physical or emotional abuse [3]. In Tanzania, a national survey with a representative sample of more than 3,700 youths between the ages of 13 and 24 revealed that almost three quarters of both girls and boys had experienced physical discipline (e.g., being punched, whipped, or kicked) and more than one quarter faced emotional violence (e.g., being insulted, humiliated, threat of abandonment) prior to the age of 18 [4]. More than half of the girls and boys aged 13–17 years reported that they had experienced physical violence by either a relative or an authority figure during the past year. Concordantly, in a recent study with primary school students aged 6 to 15, it was found that nearly all children had experienced corporal punishment at some point during their lifetime in both family and school contexts [5]. Half of the respondents reported having experienced corporal punishment within the last year. Furthermore, a study conducted at secondary schools showed that 40 % of the teachers reported the frequent use (defined as more than ten times a week) of physical discipline (e.g., beating with a stick) in school [6]. Interviews with teachers and students confirmed that caning (i.e., being beaten with a stick) was the most frequently used disciplinary method in schools. Harsh discipline seems to be a very common phenomenon in Tanzania. However, little is known about the impact of harsh discipline on the children’s mental health, cognitive functioning and school performance in societies in which these disciplinary methods are commonly utilized.

### Relation between harsh discipline, child abuse and mental health problems

Prior research, mostly from Western countries, showed that in addition to physical injury, child maltreatment is associated with a number of emotional and behavioral problems that begin in childhood but may last through adolescence and adulthood [7]. Adverse effects of child abuse include depression, anxiety disorders, substance abuse, and aggressive or delinquent behavior [8–10]. Physical abuse, for example, contributed significantly to other risk factors in accounting for lifetime diagnoses of major depression, conduct disorder, and drug abuse [11, 12]. Corporal punishment also shows an association with psychopathology. Individuals who had experienced corporal punishment were at increased odds of psychopathology compared to those who had not experienced corporal punishment [13]. Both cross-sectional and longitudinal studies suggested that corporal punishment is associated with increased externalizing and internalizing problems in childhood, adolescence and adulthood [14–18].

Psychological or emotional maltreatment also seems to be a potent form of maltreatment that has been linked with attachment disorders, developmental problems, aggression, and later psychopathology [19, 20]. For example, emotional abuse was associated with later symptoms of anxiety and depression [21] and parental verbal aggression with depression, anxiety and anger-hostility [22].

### Harsh discipline, child abuse and its impact on cognitive functioning

Yet, child maltreatment is not only risk factors for mental health problems, it may also affect the cognitive functioning [23]. For example, child abuse is related to delayed language and cognitive development, lower IQ and poorer school performance [24]. A systematic review showed that teenagers exposed to child maltreatment performed more poorly than nonmaltreated controls on tasks assessing working memory, attention and executive functions [25]. Augusti and Melinder [26] replicated this negative association between child maltreatment and working memory capacity. A number of studies reported not only an association of parental verbal abuse and emotional abuse with impaired spatial working memory performance but also with an alteration in the integrity of neural pathways, which seem to have implications for language development [27, 28].

Most studies investigating the link between child maltreatment and cognitive functioning have been conducted in children or adolescents who also reported mental health problems. Therefore, it is difficult to disentangle whether child maltreatment has a direct impact on cognitive functioning or whether it is mediated through mental health disorders [24]. Deficits in executive functions and working memory seem to mediate the relationship between child abuse and lowered school performance [29]. For example, physically abused children displayed an overwhelming set of problems on nearly all dimensions of school performance [30]. However, less is known about the association between harsh punishment and cognitive functioning.

Internalizing problems seem to contribute to the prediction of school performance and cognitive functioning over and above the effects of intelligence [31]. Overall, prior research findings indicated an association between child abuse and internalizing problems as well as between internalizing problems and cognitive dysfunctions. Therefore, internalizing problems may provide one route through which abused children are at heightened risk for impaired cognitive functioning and thus lowered school performance.

### Objectives

In Tanzania and other countries children are frequently exposed to harsh discipline, which may have detrimental consequences for their mental health and cognitive functioning. We have already demonstrated that corporal punishment was linked to children’s externalizing problems in Tanzanian children [5]. Yet, in our work at Tanzanian schools and child care institutions it became obvious that mainly children with externalizing problem behavior were regarded as “*problem children*”, whereas caregiver and teachers did not notice or report internalizing problems of children. Therefore, in the present study we aimed at investigating more closely children’s internalizing problems and its association with harsh discipline, children’s working memory capacity and school performance in a sample of Tanzanian primary school students. We hypothesized that (a) exposure to harsh discipline would be related to internalizing problems, and cognitive dysfunctioning (i.e., lower school performance and working memory). We also predicted that (b) internalizing problems would be negatively associated with cognitive dysfunctioning. Furthermore, we expected (c) that internalizing problems would mediate the association between harsh discipline and cognitive dysfunctioning.

## Methods

### Participants

All children participating in this study were enrolled at one primary school in a town of approximately 150,000 inhabitants in southern Tanzania. We interviewed 409 children (52 % boys) in the 2^nd^ to 7^th^ year of formal schooling, with a mean age of 10.49 years (SD = 1.89, range: 6–15). The majority of the children lived together with their families. In total, 55 % (*n* = 227) of the children reported living together in one household with their mother and their father; 11 % (*n* = 46) with their mother but not their father; 3.6 % (*n* = 15) with their father but not their mother; 11 % (*n* = 46) with other relatives (e.g., grandparents, uncle, aunt etc.) but not their parents, 16 % (*n* = 65) lived in institutional care, and 2.5 % (*n* = 10) in foster families. In total, 18 % (*n* = 73) of the children reported that one parent had died and 3.9 % (*n* = 16) that both parents had died. This seems to be comparable to previous findings from Tanzania. For example, in 2009 25 % of the girls and 20 % of the boys were orphans [4].

### Procedure

The research team consisted of five German psychologists and seven Tanzanian psychologists and community workers. The interviewers were trained in interview skills, conducting interviews with children, and in the concepts of internalizing problems and working memory. They were also trained in the translation of the instruments from English to Swahili and the translation of the participants’ responses from Swahili to English for the German psychologists. The interviewers received instruction for these skills during a 2-week training session that included role-plays and interview observation. The project leaders were present throughout the entire training and data collection phases and supervised the research team throughout all stages of the study. The interviewers had standardized the form of assessment by conducting joint and double-rated interviews. In the total sample, 33 interviews were double-rated by two independent assessors to determine inter-rater reliability (see below). By following established international guidelines [32], all instruments were translated in written form to Swahili and were intensely discussed to guarantee a precise translation prior to data collection. A written, blind back-translation into English ensured valid and accurate translation. One of the authors (TH) speaks Swahili fluently and thus could ensure valid translation.

Before data collection we sent a letter and a written informed consent form to all parents or caregivers explaining the purpose of the study. The letter clarified that the participation of the children would be entirely voluntary, no monetary compensation would be offered, and invited them to call or meet the project leaders in case of additional questions. Approximately 80 % of the parents and caregivers signed the informed consent and sent it back. Every child was interviewed individually in a private setting. The children were assured that the interview was confidential and that they were free to end the interview at any time. The interviews each took 1.5 h on average.

### Measures

All interviews were conducted in Swahili and administered as a structured interview. In this way, young children could also be interviewed using all instruments. The first part of the interview consisted of socio-demographic information including age, grade and sex.

#### Harsh punishment

Harsh punishment was measured using the Maltreatment and Abuse Chronology of Exposure - Pediatric Version (pediMACE; (Isele, Hecker, Hermenau, Elbert, Ruf-Leuschner, Moran, Teicher, and Schauer: Assessing exposure to adversities in children: The pediatric Maltreatment and Abuse Chronology of Exposure Interview, submitted)) [33]. The pediMACE consists of 45 dichotomous (yes/no) questions, measuring witnessed or self-experienced types of child maltreatment throughout the lifetime with a particular focus on family violence. The pediMACE has been successfully validated with children in Tanzania (Isele et al.: Assessing exposure to adversities in children: The pediatric Maltreatment and Abuse Chronology of Exposure Interview, submitted). For our analyses, we included only the questions covering possible forms of harsh emotional and physical discipline by an adult person (e.g., parent, relative or caregiver) living in the same household (see Table 1). The interviewer rated the child’s report as never happened in life (0) or happened at least one time (1). For further analysis, we calculated separate scores for emotional and physical discipline by totaling up all of the relevant question responses. Four questions were related to physical discipline. Cohen’s *k* was > .99 (.99 – 1). On average the children reported exposure to M = 2.29 (SD = 1.07; range: 0 – 4) different forms of physical discipline. Emotional discipline was assessed with three questions. Cohen’s *k* was > .99 (.99 – 1). Participants have been exposed to M = 1.30 (SD = 0.74; range: 0 – 3) different forms of emotional discipline.

**Table 1:** Occurrence of harsh discipline during the children’s lifetime (*N* = 409)

	% (n)
Physical discipline	
1) Has an adult in your household intentionally pinched, slapped, punched or kicked you?	66 (270)
2) Has an adult in your household spanked you with the palm of his/her hand on your buttocks, arms or legs?	57 (231)
3) Has an adult in your household spanked you with an object such as a strap, belt, stick, tube, broom, wooden spoon?	82 (336)
4) Has an adult in your household hit you so hard that you were injured?	24 (99)
Emotional discipline	
1) Has an adult in your household called you names or said hurtful things (e.g., fat, ugly, stupid)?	41 (169)
2) Has an adult in your household yelled or screamed at you?	82 (336)
3) Has an adult in your household locked you in a dark and narrow place (e.g., basement, closet)?	7 (27)

#### Internalizing problems

The self-evaluation of internalizing problems was assessed with the Strengths and Difficulties Questionnaire (SDQ) [34]. The SDQ comes with good psychometric properties and has been utilized internationally [35]. It has also been successfully implemented in Tanzanian settings [36, 37]. It consists of 25 statements with corresponding response categories of *not true (0)*, *somewhat true (1)* or *certainly true (2)*. In the present sample the Cronbach’s α was .67 and the Cohen’s *k* was .99 (.94 – 1). We focused on the subscales measuring internalizing problems: the peer problems scale and emotional symptoms scale. Both subscales have a possible range from 0 to 10. In the peer problem scale a score of 4 to 5 indicates an enhanced level of peer problems and a score higher than 5 an abnormal level. In the emotional symptoms scale a score of 6 indicates an enhanced level of emotional symptoms and a score higher than 6 an abnormal level.

Furthermore, we assessed the severity of depressive symptoms by means of the Children’s Depression Inventory (CDI) [38, 39]. The CDI is a reliable and well-tested clinical research instrument designed for school-aged children and adolescents. It has been successfully implemented in Tanzanian settings [40]. It evaluates the severity of specific depressive symptoms and contains 27 items with three statements each and the child has to choose which statement fits best. For each item, the points range from 0 to 2, where higher values represent more clinically severe symptoms. In the present sample the Cronbach’s α was .70 and the Cohen’s *k* was .99 (.92 – 1). A cutoff point of 12 has been established as the ideal threshold discriminating children at risk of depression from nondepressed children [38, 40].

#### Cognitive functioning

As measures of cognitive functioning we assessed school performance and working memory capacity. We chose school performance as one proxy for cognitive functioning as (1) it can be measured in the daily environment of the children, (2) it is an objective measure that is not influenced by the specific circumstances of the study and (3) it is an others-report measure. We assessed the school performance using the results of the midterm exams (0 – 100 %) in the four core subjects during the period of assessment: Mathematics, English, Swahili and Science. We received the results from the school administration. In order to control for possible influences by teachers, grade or dynamics within the classes, we z-standardized the grades within each class. The z-standardized scores ranged in the four main subjects as follows: English (range: −2.95 – 2.29), Mathematics (range: −2.80 – 2.92), Swahili (range: −3.40 – 1.81), and Science (range: −3.05 – 2.09).

Working memory capacity as a measure of cognitive functioning was used because (1) it was independent of the educational level and experience with technical equipment (e.g., computers, tablets or smartphones) of the students, (2) the assessment of working memory capacity includes other cognitive functions (e.g., attention and executive functions) and (3) it allowed a feasible and economic assessment of cognitive functioning in the context of a Tanzanian school. We measured working memory capacity with the Corsi Block-Tapping Task [41]. It has been utilized as a measure of spatial memory in both clinical and experimental contexts for several decades, is the most important nonverbal task in neuropsychological research, and comes with good psychometric properties [42, 43]. The task requires the children to reproduce block-tapping sequences of increasing length in the same or in the reversed order and provides an index of working memory capacity. The Corsi apparatus consisted of nine 2.25 cm^3^ black, wooden blocks fixed to a 27.5 cm × 22.8 cm grey, wooden board. The blocks were cubes placed as described in the original test developed by Corsi [41]. Each cube was numbered on one side so that the numbers were visible to the interviewer but not to the participant. The participant was seated in front of the interviewer, who subsequently tapped the blocks starting with a sequence of three blocks. Three trials were given per block sequence of the same length. The blocks were touched with the index finger at a rate of approximately 1 block per second with no pauses between the individual cubes. In the first application of the test after the first half of the interview the participant had to tap the block sequences in the same order immediately after the interviewer was finished. In the second application in the end of the interview the participants had to tap the block sequence in reversed order. We introduced the test as a game and used the following instruction: *“My finger is a monkey and he will jump from tree to tree. When the monkey has finished jumping, I want you to follow the monkey in the same way.*” In the second application, we used a slightly different instruction: “*My finger is a monkey. He will jump from tree to tree but this time he has forgotten his way back. Can you show the monkey the way back*?” We computed a total score for each application (same order and reversed order) by multiplying the memory span (length of the last sequence that has been correctly repeated twice) and the number of correctly repeated sequences until the test was discontinued (i.e., the number of correct trials). The total score ranged from 0 to 189. This score is more reliable than memory span alone [44]. On average, the participants reported a total score on the first application (same order) of *M* = 37.50 (*SD* = 17.36, range: 0 – 105) and on the second application (reversed order) of *M* = 29.32 (*SD* = 18.70, range: 0 – 98).

### Data analysis

We conducted SEM analyses applying maximum likelihood method of estimation [45]; in case of missing data, means and intercepts were estimated. As we aimed to test the association of harsh discipline with different outcome variables, we excluded all children (*n* = 21) from our SEMs who reported exposure to any form of sexual abuse in their home (measured with pediMACE). Furthermore, for logistical reasons, seven interviews could not be completed and five children did not participate in the mid-term exams.

Since we aimed to model the effects of different manifest variables on latent variables at a given level of generality, parceling items and the use of sum scores in the SEM is warranted [46]. Goodness of fit was assessed using the following indices: chi-square (χ^2^); confirmatory fit index (CFI), with values greater than .95 indicating good fit; and root mean square error of approximation (RMSEA), with values less than .08 indicating reasonable fit [47, 48]. Akaike Information Criterion (AIC) is a comparative measure of fit. When comparing AIC values, *exp((AIC*_*min*_*- AIC*_*i*_*)/2)* can be interpreted as the relative probability that the *i*th model minimizes the (estimated) information loss [49]. Preliminary analyses confirmed that all statistical assumptions (normality, linearity, collinearity, reliability and missing value analysis) for using SEM were met. Neither univariate nor multivariate outliers could be detected. All measurement models showed reasonable fit. Inter-correlations are displayed in Additional file 1: Table S1. Our metric for a small effect size was β ≥ .10, for a medium effect β ≥ .30, and for a large effect β ≥ .50 [50]. Data was analyzed with IBM SPSS Statistics Version 21 for MAC and IBM SPSS Amos Version 21 for Windows.

## Results

### Internalizing problems

In the peer problem scale the children showed, on average, a score of M = 2.09 (SD = 1.63; range: 0 – 8). In total, 15 % (*n* = 60) of the children showed an enhanced level of peer problems and 8 % (*n* = 33) an abnormal level. In the emotional symptoms scale the children showed on average a score of M = 3.19 (SD = 2.31; range: 0 – 10). In total, 9 % (*n* = 35) of the children showed an enhanced level of emotional symptoms and 9 % (*n* = 37) an abnormal level. Participants reported an average CDI score of *M* = 6.76 (*SD* = 4.57, range: 0 – 25). In total, 14 % (*n* = 57) of the children reported a CDI score of 12 or higher and thus were considered to be at risk of depression.

### Associations between harsh punishment, internalizing problems and cognitive functions

In the first SEM, we tested the direct association between *harsh discipline* and *internalizing problems, working memory* as well as *school performance*. The results of the first SEM analysis indicated that the hypothesized model showed reasonable model fit (χ^2^[40, *n* = 388] = 69.32 (*p* = .003), RMSEA = .044 [90 %-CI = .025–.060, PClose = .718], CFI = .968, ACI = 143.32). All manifest variables loaded significantly on the latent variables (see Table 2). *Harsh discipline* correlated significantly with *internalizing problems* indicating a large effect. However, in contrast to our hypothesis *harsh discipline* was neither directly related to *working memory* nor to *school performance. Working memory* correlated positively with school performance indicating a medium effect.

**Table 2:** Maximum likelihood estimates of the first SEM

	Unst. est.	SE	St. est.	CR
Harsh discipline				
Physical discipline	1.37	0.30	.82	4.63***
Emotional discipline	1.00	-	.70	-
Internalizing problems				
Harsh discipline	1.45	0.33	.50	4.35***
Emotional symptoms (SDQ)	1.00	-	.62	-
Peer problems (SDQ)	0.52	0.09	.45	5.64***
Depressive symptoms (CDI)	1.91	0.32	.61	5.99***
Working memory				
Harsh discipline	−2.23	2.19	-.08	−1.02
Block-Tapping test forward	1.00	-	.83	-
Block-Tapping test backward	0.92	0.20	.71	4.59***
School performance				
Harsh discipline	−0.01	0.09	-.01	−0.12
Working memory	0.01	0.03	.31	3.80***
School grades English	1.22	0.11	.74	10.97***
School grades Mathematics	1.14	0.11	.70	10.56***
School grades Swahili	1.00	-	.61	-
School grades Science	1.35	0.12	.83	11.51***

*n* 388, *Unst. est* unstandardized maximum likelihood estimates, *SE* standard error, *St. est.* standardized maximum likelihood estimates, *CR* critical ratio

***p ≤ .001

**Fig. 1:**
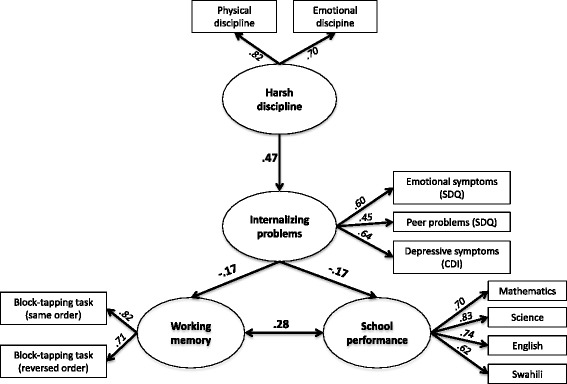
SEM testing the indirect association (via internalizing problems) between harsh discipline, working memory and school performance. This model is consistent with the view that harsh discipline aggravates internalizing problems, which in turn degrade both, working memory and school performance. Note. Standardized maximum likelihood estimates (regression weights in bold and factor loadings in italics) are depicted; all paths are significant beyond .05. Error variables are omitted for clarity. SDQ: Strength and Difficulties Questionnaire; CDI: Children’s Depression Inventory

In the second SEM, we tested the direct association between *harsh discipline* and children’s *internalizing problems* as well as the indirect association (via *internalizing problems*) between *harsh discipline* and *working memory* capacity as well as *school performance*. We decided not to include the direct paths from *harsh discipline* to *working memory* and *school performance* as these paths were not significant in Model 1. The results of the second SEM analysis indicated that the hypothesized model showed a good model fit (χ^2^[40, *n* = 388] = 59.03 (*p* = .027), RMSEA = .035 [90 %-CI = .012–.053, PClose = .909], CFI = .979, ACI = 133.03). When we compared the AIC values of both models, we found that Model 1 is 0.0058 times as probable as Model 2 to minimize the information loss. Hence, AIC indicated a better model fit for Model 2 compared to Model 1. All manifest variables loaded significantly on the latent variables (see Table 3). *Harsh discipline* correlated significantly with *internalizing problems* indicating a large effect. Furthermore, *internalizing problems* were negatively related to both *working memory* and *school performance* indicating in each case a small effect. *Working memory* correlated positively with school performance indicating a medium effect (see Fig. 1).

**Table 3:** Maximum likelihood estimates of the second SEM

	Unst. est.	SE	St. est.	CR
Harsh discipline				
Physical discipline	1.38	0.31	.82	4.41***
Emotional discipline	1.00	-	.70	-
Internalizing problems				
Harsh discipline	1.34	0.32	.47	4.18***
Emotional symptoms (SDQ)	1.00	-	.60	-
Peer problems (SDQ)	0.53	0.09	.45	5.74***
Depressive symptoms (CDI)	2.04	0.33	.64	6.18***
Working memory				
Internalizing problems	−1.70	0.78	-.17	−2.18*
Block-Tapping test forward	1.00	-	.82	-
Block-Tapping test backward	0.94	0.20	.71	4.77***
School performance				
Internalizing problems	−0.07	0.03	-.17	−2.27*
Working memory	0.01	0.01	.28	3.62***
School grades English	1.21	0.11	.74	11.01***
School grades Mathematics	1.14	0.11	.70	10.62***
School grades Swahili	1.00	-	.62	-
School grades Science	1.34	0.12	.83	11.57***

*n* 388*, Unst. est* unstandardized maximum likelihood estimates, *SE* standard error, *St. est.* standardized maximum likelihood estimates, *CR* critical ratio

*p ≤ .05. ***p ≤ .001

## Discussion

Our results indicated that 14 % of the participating students reported clinically relevant symptoms of depression in a common screening instrument (CDI). Using the SDQ 8 % respectively 9 % reported clinically relevant levels of peer problems (e.g., social withdrawal) and emotional problems. These numbers indicate that internalizing problems are also of relevance in Tanzanian primary school students though neither teachers nor caregivers reported internalizing problem behavior of children during the preparatory phase of our study. These findings may explain why so many parents, caregivers and teachers in Tanzania and elsewhere are convinced that harsh discipline does no harm to the children: Often the children suffer in silence and neither their parents, their caregivers nor their teachers notice the children’s suffering.

Harsh discipline may impair mental health across the entire lifespan [13]. In line with this, we found a strong relationship between harsh discipline and internalizing problems. In turn, internalizing problems were related to lower working memory capacity and lower school performance. However, in contrast to our hypothesis the extent of harsh discipline was not directly linked to impaired working memory capacity and poor school performance. Our findings indicate that harsh discipline may not only be related to poor mental health but may also be associated with reduced cognitive functioning via internalizing mental health problems. Our results are in concordance with prior studies mostly from Western countries suggesting a relationship between exposure to child abuse, including harsh discipline, and internalizing problems [9, 22] and cognitive functions [27, 28]. However, it is not yet clear whether harsh discipline directly impacts cognitive functions or whether mental health problems mediate this relationship [24]. The results of the present study suggest that internalizing problems may provide one route through which harshly disciplined children are at risk for both impaired memory functions and for lowered school performance.

The effect size of this association implies a small but marked influence. These results are congruent with previous research indicating a cumulative effect of harsh discipline on the children’s mental health problems [5, 14, 22]. Considering the high prevalence of harsh discipline in the current sample and other reports with representative samples [1, 2, 4], the consequences of harsh discipline may manifest into a considerable cause for concern at the societal level [1]. Yet, further research is needed to thoroughly understand the causal mechanisms that may underlie the relationship between harsh discipline, mental health problems and cognitive deficits. Particularly, further studies that examine these relations across countries and societies in which harsh disciplinary methods are legal and highly prevalent using scientific rigorous designs are essential.

The findings of the present study emphasize the need to inform parents, caregivers, governmental organizations, and the population at large, especially in countries with a high prevalence rate of harsh discipline, about the potentially adverse consequences associated with harsh discipline. Thereby, it is important to raise awareness that children also suffer from internalizing problems (e.g., depressive symptoms, social withdrawal, etc.) helping parents, caregivers and teachers to be able to notice also a quiet child that suffers from mental health problems in order to break the vicious cycle that our results may indicate: Children suffer in silence, their suffering is not noticed but as a result of poor school performance these children may be even more likely to experience further harsh punishment.

Furthermore, our findings suggest that effective prevention of harsh discipline may be required to help to prevent children from developing mental health problems. Beside economic and environmental factors (e.g., poverty, high stress level, societal beliefs), reasons for using harsh disciplinary methods that parents and caregivers reported to researchers were, among others, a lack of non-violent caregiving skills, excessive demands, and helplessness [51]. Lansford et al. [52] showed that parents’ positive evaluations of aggressive responses to hypothetical childrearing vignettes predicted parents’ self-reported harsh discipline in a study in nine countries. Thus, efforts to eliminate harsh discipline toward children could target parents’ beliefs about the acceptability of using harsh discipline. Hermenau et al. [51] successfully tested the feasibility of a preventative approach with caregivers in Tanzania that focused on caregivers’ beliefs, self-reflection of own experiences of harsh discipline, positive parenting skills, and nonviolent caregiving strategies. We recommend more research efforts that focus on developing and testing culturally appropriate prevention programs that effectively replace harsh discipline by forms of educational measures that do not harm the children.

There are some limitations of the study that should be noted: first, the cross-sectional study design does not allow for the establishment of causality. We cannot, for example, completely rule out the possibility that children who perform poorly at school experienced more harsh discipline and thus develop mental health issues. However, in our models we did not find a direct association between poor school performance and harsh discipline. The recruitment of study participants at one primary school limits the generalizability of our findings. In the school context of our data assessment we were unable to include parents’ reports for logistical reasons. Therefore, we could not gather information regarding the socio-economic status (SES) of our sample. It remains to be tested whether SES may impact cognitive functioning through other pathways than harsh discipline. Generally, the children talked very openly about their experiences and feelings. However, potential biases, such as social desirability, can never be completely ruled out for subjective reports. However, a recent study strengthened the credibility of children’s self-report by comparing epigenetic and self-report data and supported the conclusion that children are capable of accurately reporting their exposure to abuse [53]. Furthermore, we did not assess the exposure to harsh discipline by teachers systematically. The exposure to harsh discipline in school may have further added to the impact of harsh discipline at home.

## Conclusions

The present study suggests that harsh discipline is closely linked to children’s internalizing problems, which are in turn associated with lower cognitive functioning and school performance. Given the high rates of harsh discipline experienced by children in East African homes and elsewhere, the findings of the present study emphasize the need to inform the population at large about the potentially adverse consequences associated with harsh discipline. Further, our findings underscore the need to implement preventative measures against the use of these forms of discipline. Through these efforts, reducing harsh discipline in their home environments combined with the fostering of positive caregiving skills we would enable children to grow up in a respectful and supportive atmosphere, thereby strengthening their development.

## Ethics and consent to participate

The Tanzanian Commission for Science and Technology and the Ethical Review Board of the University of Konstanz approved the study. Only children with an informed consent signed by their parents or caregivers were included in this study.

## Consent to publish

Not applicable.

## Availability of data and materials

All data are fully available without restriction from the corresponding author.

## Additional file

Additional file 1: Table S1. Inter-correlations of all relevant variables. (DOCX 15 kb)

## Abbreviations

AIC: Akaike information criterion
CDI: children’s depression inventory
CFI: confirmatory fit index
IQ: intelligence quotient
pediMACE: maltreatment and abuse chronology of exposure - pediatric version
RMSEA: root mean square error of approximation
SDQ: strengths and difficulties questionnaire
SEM: structural equation model
SES: socio-economic status
SPSS: statistical package for the social sciences

## References

[1] Straus MA (2010). Prevalence, societal causes, and trends in corporal punishment by parents in world perspective. Law Contemp Probl.

[2] UNICEF (2010). Child disciplinary practices at home: Evidence from a range of Low- and Middle-Income Countries.

[3] Leeb RT, Paulozzi L, Melanson C, Simon T, Arias I. Child Maltreatment Surveillance: Uniform Definitions for Public Health and Recommended Data Elements. Atlanta, GA: Centers for Disease Control and Prevention, National Center for Injury Prevention and Control; 2008

[4] UNICEF (2011). Violence against children in Tanzania: Results from a National Survey 2009.

[5] Hecker T, Hermenau K, Isele D, Elbert T (2014). Corporal punishment and children’s externalizing problems: a cross-sectional study of Tanzanian primary school students. Child Abuse Negl.

[6] Feinstein S, Mwahombela L (2010). Corporal punishment in Tanzania’s schools. Int Rev Educ.

[7] Carr CP, Martins CMS, Stingel AM, Lemgruber VB, Juruena MF (2013). The role of early life stress in adult psychiatric disorders: a systematic review according to childhood trauma subtypes. J Nerv Ment Dis.

[8] Dube SR, Felitti VJ, Dong M, Chapman DP, Giles WH, Anda RF (2003). Childhood abuse, neglect, and household dysfunction and the risk of illicit drug use: the adverse childhood experiences study. Pediatrics.

[9] Sugaya L, Hasin DS, Olfson M, Lin K, Grant BF, Blanco C (2012). Child physical abuse and adult mental health: a national study. J Trauma Stress.

[10] Hermenau K, Hecker T, Elbert T, Ruf-Leuschner M (2014). Maltreatment and mental health in institutional care – comparing early- and late-institutionalized children in Tanzania. Infant Ment Health J.

[11] Kaplan SJ, Pelcovitz D, Salzinger S, Weiner M, Mandel FS, Lesser ML, Labruna VE. Adolescent physical abuse: risk for adolescent psychiatric disorders. Am J Psychiatry. 1998;155:954–9. 10.1176/ajp.155.7.954 9659863

[12] Widom CS, DuMont K, Czaja SJ (2007). A prospective investigation of major depressive disorder and comorbidity in abused and neglected children grown up. Arch Gen Psychiatry.

[13] Afifi TO, Brownridge DA, Cox BJ, Sareen J (2006). Physical punishment, childhood abuse and psychiatric disorders. Child Abuse Negl.

[14] Gershoff ET, Lansford JE, Sexton HR, Davis-Kean P, Sameroff AJ (2012). Longitudinal links between spanking and children’s externalizing behaviors in a national sample of White, Black, Hispanic, and Asian American families. Child Dev.

[15] Coley RL, Kull MA, Carrano J (2014). Parental endorsement of spanking and children’s internalizing and externalizing problems in African American and Hispanic families. J Fam Psychol.

[16] Maguire-Jack K, Gromoske AN, Berger LM (2012). Spanking and child development during the first 5 years of life. Child Dev.

[17] McKee L, Roland E, Coffelt N, Olson AL, Forehand R, Massari C, Jones D, Gaffney CA, Zens MS. Harsh discipline and child problem behaviors: the roles of positive parenting and gender. J Fam Violence. 2007;22:187–96.

[18] Turner HA, Finkelhor D (1996). Corporal punishment as a stressor among youth. J Marriage Fam.

[19] Hibbard R, Barlow J, MacMilan H, Committee on Child Abuse and Neglect, American Academy of Child and Adolescent Psychiatry CM and VC (2012). Clinical report: psychological maltreatment. Pediatrics.

[20] Iffland B, Sansen LM, Catani C, Neuner F (2012). Emotional but not physical maltreatment is independently related to psychopathology in subjects with various degrees of social anxiety: a web-based internet survey. BMC Psychiatry.

[21] Wright MOD, Crawford E, Del Castillo D (2009). Childhood emotional maltreatment and later psychological distress among college students: the mediating role of maladaptive schemas. Child Abus Negl.

[22] Teicher MH, Samson JA, Polcari AM, Mcgreenery CE (2006). Sticks, stones, and hurtful words: relative effects of various forms of childhood maltreatment. Am J Psychiatry.

[23] Mills R, Alati R, O’Callaghan M, Najman JM, Williams GM, Bor W, Strathearn L. Child abuse and neglect and cognitive function at 14 years of age: findings from a birth cohort. Pediatrics. 2011;127:4–10. 10.1542/peds.2009-3479 21135010

[24] Hart H, Rubia K (2012). Neuroimaging of child abuse: a critical review. Front Hum Neurosci.

[25] Irigaray TQ, Pacheco JB, Grassi-Oloveira R, Fonseca RP, de Cavalho Leite JC, Kristensen CH (2013). Child maltreatment and later cognitive functioning: a systematic review. Psicol Reflex e Crit.

[26] Augusti E-M, Melinder A (2013). Maltreatment is associated with specific impairments in executive functions: a pilot study. J Trauma Stress.

[27] Choi J, Jeong B, Rohan ML, Polcari AM, Teicher MH (2009). Preliminary evidence for white matter tract abnormalities in young adults exposed to parental verbal abuse. Biol Psychiatry.

[28] Majer M, Nater UM, Lin J-MS, Capuron L, Reeves WC (2010). Association of childhood trauma with cognitive function in healthy adults: a pilot study. BMC Neurol.

[29] DePrince AP, Weinzierl KM, Combs MD (2009). Executive function performance and trauma exposure in a community sample of children. Child Abuse Negl.

[30] Kurtz PD, Gaudin JM, Wodarski JS, Howing PT (1993). Maltreatment and the school-aged child: School performance consequences. Child Abuse Negl.

[31] Rapport MD, Denney CB, Chung KM, Hustace K (2001). Internalizing behavior problems and scholastic achievement in children: cognitive and behavioral pathways as mediators of outcome. J Clin Child Psychol.

[32] Brislin RW, Lonner WJ, Thorndike RM (1973). Cross-Cultural Research Methods.

[33] Teicher MH, Parigger A (2015). The “Maltreatment and Abuse Chronology of Exposure” (MACE) Scale for the Retrospective Assessment of Abuse and Neglect During Development. PLoS ONE.

[34] Goodman R, Meltzer H, Bailey V (1998). The strengths and difficulties questionnaire: a pilot study on the validity of the self-report version. Eur Child Adolesc Psychiatry.

[35] Goodman R, Ford T, Simmons H, Gatward R, Meltzer H (2000). Using the Strengths and Difficulties Questionnaire (SDQ) to screen for child psychiatric disorders in a community sample. Br J Psychiatry.

[36] Hermenau K, Hecker T, Ruf M, Schauer E, Elbert T, Schauer M (2011). Childhood adversity, mental ill-health and aggressive behavior in an African orphanage: changes in response to trauma-focused therapy and the implementation of a new instructional system. Child Adolesc Psychiatry Ment Health.

[37] Hermenau K, Eggert I, Landolt MA, Hecker T (2015). Neglect and perceived stigmatization impact psychological distress of orphans in Tanzania. Eur J Psychotraumatol.

[38] Kovacs M (2001). Children’s Depression Inventory (CDI): Technical Manual.

[39] Sitarenios G, Kovacs M, Maruish ME (1999). Use of the Children’s Depression Inventory. The use of psychological testing for treatment planning and outcomes assessment.

[40] Traube D, Dukay V, Kaaya S, Reyes H, Mellins C (2010). Cross-cultural adaptation of the child depression inventory for use in Tanzania with children affected by HIV. Vulnerable Child Youth Stud.

[41] Milner B (1971). Interhemispheric differences in the localization of psychological processes in man. Br Med Bull.

[42] Farrell Pagulayan K, Busch RM, Medina KL, Bartok JA, Krikorian R (2006). Developmental normative data for the Corsi Block-Tapping task. J Clin Exp Neuropsychol.

[43] Orsini A (1994). Corsi’s block-tapping test: standardization and concurrent validity with the WISC-R for children aged 11–16. Percept Mot Ski.

[44] Kessels RP, van Zandvoort MJ, Postma A, Kappelle LJ, de Haan EH (2000). The corsi block-tapping task: standardization and normative data. Appl Neuropsychol.

[45] Schumacker RE, Lomax RG (2010). A Beginner’s Guide to Structural Equation Modeling.

[46] Little TD, Cunningham WA, Shahar G, Widaman KF (2002). To Parcel or Not to Parcel: Exploring the Question, Weighing the Merits. Struct Equ Model A Multidiscip J.

[47] Hu L, Bentler PM (1999). Cutoff criteria for fit indexes in covariance structure analysis: conventional versus new alternatives. Struct Equ Model.

[48] Kline RB (2005). Principles and Practice of Structural Equation Modeling.

[49] Burnham KP, Anderson DR (2002). Model Selection and Multimodel Inference: A Practical Information-Theoretic Approach.

[50] Shrout PE, Bolger N (2002). Mediation in experimental and nonexperimental studies: New procedures and recommendations. Psychol Methods.

[51] Hermenau K, Kaltenbach E, Mkinga G, Hecker T (2015). Improving care quality and preventing maltreatment in institutional care – a feasibility study with caregivers. Front Psychol.

[52] Lansford JE, Sharma C, Malone PS, Woodlief D, Dodge KA, Oburu P, Pastorelli C, Skinner AT, Sorbring E, Tpanya S, Tirado LM, Zelli A, A--Hassan S, Alampay LP, Bacchini D, Bombi AS, Bornstein MH, Chang L, Deater-Deckard K, Di Giunta L. Corporal punishment, maternal warmth, and child adjustment: a longitudinal study in eight countries. J Clin Child Adolesc Psychol. 2014;43:670–85. 10.1080/15374416.2014.893518 PMC4101150 24885184

[53] Hecker T, Radtke K, Hermenau K, Papassotiropoulos A, Elbert T. Associations between child abuse, mental health and epigenetic modifications in the proopiomelanocortin gene (POMC): a study with children in Tanzania. Dev Psychopathol. 2016. doi:10.1017/S0954579415001248. Advance online publication 10.1017/S0954579415001248 26753719

