# Supplementary material for: Harsh discipline relates to internalizing problems and cognitive functioning: findings from a cross-sectional study with school children in Tanzania
Source: BMC Psychiatry. 2016 Apr 29;16:118. doi: 10.1186/s12888-016-0828-3 (PMC4850652; doi:10.1186/s12888-016-0828-3)
Supplement: Additional file 1: Table S1. — Inter-correlations of all relevant variables. (DOCX 15 kb) [file 12888_2016_828_MOESM1_ESM.docx]

Supplementary Table A

*Inter-correlations of all relevant variables (N = 409)*

|  | 1 | 2 | 3 | *4* | 5 | 6 | 7 | 8 | 9 | 10 | 11 |
| --- | --- | --- | --- | --- | --- | --- | --- | --- | --- | --- | --- |
| 1. Physical discipline | 1 |  |  |  | . |  |  |  |  |  |  |
| 2. Emotional discipline | .44*** | 1 |  |  |  |  |  |  |  |  |  |
| 3. Emotional symptoms (SDQ) | .19*** | .21*** | 1 |  |  |  |  |  |  |  |  |
| 4. Peer problems (SDQ) | *.10* | .18*** | .28*** | 1 |  |  |  |  |  |  |  |
| 5. Depressive symptoms CDI) | 22*** | .20*** | .37*** | .28*** | 1 |  |  |  |  |  |  |
| 6. Block-Tapping Task forward | -.07 | .01 | -.06 | -.07 | -.09 | 1 |  |  |  |  |  |
| 7. Block-Tapping test backward | .01 | -.02 | -.04 | -.09 | -.08 | .59*** | 1 |  |  |  |  |
| 8. School grades English (z-standardized) | <.01 | .03 | -.08 | -.07 | -.07 | .22*** | .14** | 1 |  |  |  |
| 9. School grades Math (z-standardized) | -.03 | -.02 | -.05 | -.12* | -.16*** | .19*** | .24*** | .50*** | 1 |  |  |
| 10. School grades Swahili (z-standardized) | -.01 | -.01 | -.15** | -.10 | -.12* | .13** | .13** | 45*** | .48*** | 1 |  |
| 11. School grades Science (z-standardized) | <-.01 | .09 | -.11* | -.04 | -.16** | .17*** | .13** | .63*** | .58*** | .49*** | 1 |

Note*.* Correlation coefficient: Pearson’s r; **p* ≤ .05, ***p* ≤ .01, *** *p* ≤ .001
